# Thoracoscopic lung metastasectomies: a 10-year, single-center experience

**DOI:** 10.1007/s00464-012-2691-8

**Published:** 2013-01-24

**Authors:** Felice Lo Faso, Luciano Solaini, Rosalba Lembo, Paolo Bagioni, Silvia Zago, Paolo Soliani, Robert D. Pascotto

**Affiliations:** 1 Thoracic Surgery Unit, ASL Ravenna, Maria Cecilia Hospital, Via Corriera 1, 48010 Cotignola, Ravenna Italy; 2 Department of Cardiothoracic and Intensive Care, Istituto Scientifico S. Raffaele, Via Olgettina 60, 20132 Milan, Italy; 3 Department of Pathology, ASL Ravenna, Ospedale S. Maria delle Croci, Viale Randi 5, 48121 Ravenna, Italy; 4 Heart to Heart Mission, 6910 Old Whiskey Creek Drive, Fort Myers, FL 33919 USA

**Keywords:** Pulmonary metastasis, Surgical resection, VATS surgery, Morbidity, Minimally invasive, CT scan technology

## Abstract

**Introduction:**

The ideal surgical treatment for pulmonary metastasectomy remains controversial. Minimally invasive surgery may offer advantages for quality of life outcomes, with equivalent oncologic long-term results. The purpose of our study was to confirm the validity of the thoracoscopic approach for pulmonary metastasectomy.

**Methods:**

We retrospectively reviewed 164 patients who underwent 212 lung metastasectomies from January 2000 to December 2010. Complete curative pulmonary resections were performed in 159 (96.95 %) cases; 126 patients developed lung metastases from epithelial tumors: 28 from sarcoma, 7 from melanoma, and 3 from germ cell tumors. The mean disease-free interval (DFI) was 38.75 months. Fifty-four patients underwent a major VATS resection (53 thoracoscopic lobectomies and 1 pneumonectomy), and 110 patients underwent a wedge resection/segmentectomy. Lymph node sampling was performed in 117 cases.

**Results:**

After a mean follow-up of 38 months, 87 patients (53 %) had died. All resection margins were tumor-free at final pathological examination. Multivariate analysis not confirmed in our series a better prognosis for patients with a particular histologic type and also DFI, age, number of metastases, and type of surgery did not statistically influence long-term survival.

**Conclusions:**

Thoracoscopic surgery is an acceptable procedure, safe and efficacious, with a 5-year overall survival that is equivalent to open surgery.

In recent years, lung metastases have been considered an area of interest in many discussions between surgeons to identify the best surgical approach [1]. An important study that assessed the long-term results of pulmonary metastasectomy was based on the International Registry of Lung Metastases [2]. This large registry study revealed that complete resectability, disease-free interval (DFI), and number of metastases were independent prognostic factors. Today, the majority of surgeons prefer metastasectomy by open approach to achieve bimanual lung palpation and to find and resect undetected lesions [3, 4]. In fact, the reliance on computed tomographic (CT) scanning to identify all pulmonary metastases has been criticized [5], and many surgeons prefer an open surgical approach. However, latest generation of CT scanners is superior for the detection of metastatic pulmonary nodules [6]; however, the clinical significance of these nonimaged, resected malignant nodules is unknown, and it is unclear whether the resection of these nodules improves survival. Based on these data, others surgeons use video-assisted thoracoscopic surgery (VATS) or robotic techniques for pulmonary metastasectomy, saving the patient the morbidity of thoracotomy and offering advantages in quality of life, probably with equivalent oncologic outcomes.

We started to perform VATS surgery in 1992 for the treatment of all the thoracic diseases, and thoracoscopic surgery had been favorably performed on patients who had a solitary or multiple lesions. Generally, the pulmonary metastasis was resected by pulmonary wedge resection, when it was located in the periphery of the lung, and by segmentectomy or lobectomy, when it was deep in the parenchyma [7, 8].

In this study, performed in a single-center in the past 10 years, we review our experience in the thoracoscopic surgical approach of lung metastases to evaluate whether a less invasive approach is advisable in these patients, in terms of resectability, long-term survival, and perioperative course.

## Patients and methods

We retrospectively reviewed 164 patients who underwent to 212 thoracoscopic lung metastasectomies with curative intent from January 2000 to December 2010 at the Thoracic Surgery Unit of ASL Ravenna, Ravenna, Italy, and data were retrieved from individual medical records. All patients included in the analysis had the following criteria: the primary tumor is controlled (or controllable); there is no other distant disease; complete resection of pulmonary lesions is achievable with adequate pulmonary reserve; and there are no effective medical therapies. Surgeries performed for incomplete resection, biopsy-only, and/or other diagnostic purpose were excluded.

Patients were considered eligible for curative surgery on the basis of traditional staging with chest radiograph, bronchoscopy, and thoracic/abdominal/brain CT. All 164 patients were investigated by CT scan, performed not later than 30 days from the surgery, and 84 patients also were evaluated by positron emission tomography (PET)/CT with fluorodeoxyglucose.

DFI was defined as the time between treatment of the primary tumor and the diagnosis of metastases. Ninety-seven patients had a single-lung metastasis and 67 had multiple lesions. We had three groups with different number of lesions (Table 1).

**Table 1:** Clinical characteristics

Predictor variables	Overall (%)	Alive (%)	Death (%)	*P* value
No. of patients	164 (100)	77 (46.95)	87 (53.05)	
Sex				
Male	100 (60.98)	48 (62.34)	52 (59.77)	0.7
Female	64 (39.02)	29 (37.66)	35 (40.23)	
Age (mean ± SD)	64.17 ± 14.4	65.18 ± 13.98	63.27 ± 14.78	0.4
Histology				
Sarcoma	28 (17.07)	11 (14.29)	17 (19.54)	0.2
Melanoma	7 (4.27)	4 (5.19)	3 (3.45)	
Germ cell tumors	3 (1.83)	0	3 (3.45)	
Epithelial	126 (76.83)	62 (80.52)	64 (73.56)	
Colorectal	99 (78.57)	51 (82.26)	48 (75)	0.4
Breast	14 (11.11	5 (8.06)	9 (14.06)	
Urothelial	3 (2.38)	1 (1.61)	2 (3.13)	
Gynecological	2 (1.59)	2 (3.23)	0	
Head-neck	8 (6.35)	3 (4.84)	5 (7.81)	
Metastasis				
1	97 (59.15)	44 (57.14)	53 (60.92)	0.3
2–3	50 (31.71)	28 (36.36)	24 (27.59)	
4+	17 (9.15)	5 (6.49)	10 (11.49)	
Surgery				
Wedge/segmentectomy	136 (68)	55 (71.43)	55 (63.22)	0.2
Lobectomy	54 (32.93)	22 (28.57)	32 (36.78)	
Procedure				
Single procedure	143 (87.2)	69 (89.91)	74 (85.06)	0.4
Redo	8 (10.39)	13 (14.94)	21 (12.8)	
Nodal status				
Negative	95 (57.93)	51 (66.23)	44 (50.57)	0.09
Positive	20 (12.2)	6 (7.79)	14 (16.09)	
Unknown	49 (29.88)	20 (25.97)	29 (33.3)	

We performed a total of 212 thoracoscopic metastasectomies with 54 thoracoscopic lobectomy and 158 thoracoscopic wedge resections/segmentectomy. Lymph node sampling was obtained in 117 cases.

### Surgical technique

All patients were operated on under general anesthesia with double-lumen endotracheal intubation. All were positioned in the lateral decubitus and in most cases, three ports were sufficient to achieve the thoracoscopic resection: one port for a 10-mm, 30° thoracoscope and two working ports for endoscopic instruments (endoscopic stapler or ultrasonic dissector). For major pulmonary resections, a utility mini-thoracotomy (4–5 cm) without rib spreader was added. All specimens were placed in an endoscopy bag and retrieved through anterior port or the minithoracotomy [7, 8].

### Statistical analysis

Patient characteristics are reported as means ± standard deviations or *n* (%), as appropriate. Continuous variables of patients with discordance and those without were compared with independent *t* tests. The chi-squared or Fisher’s exact test was used for categorical variables.

Overall survival (OS) was defined as the time from date of surgery to death due to any cause. The Kaplan-Meier method was used to estimate survival curves.

Univariate and subsequent multivariate binary logistic regression analyses were performed to identify independent factors. Hazard ratios (HRs) and corresponding 95% confidence intervals (CIs) also are indicated. A two-sided *P* value <0.05 was considered significant. All statistical analyses were performed with the STATA software (version 11.1; Texas USA).

## Results

**Fig. 1:**
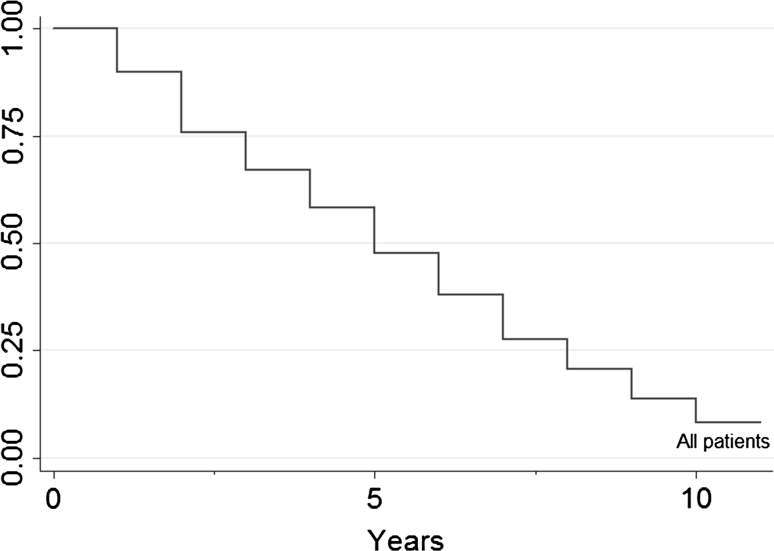
Patients survival

**Fig. 2:**
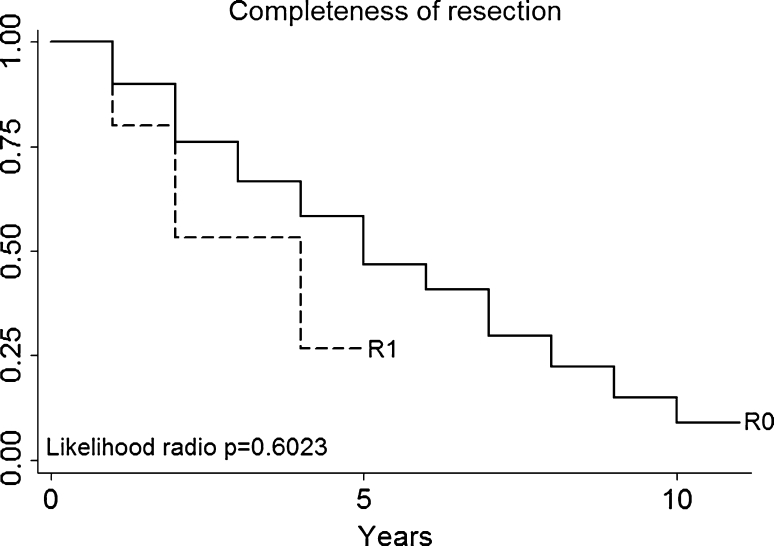
Completeness of resection

After a mean follow-up of 38 months, 77 (47 %) patients are alive (Fig. 1). Complete curative resection was obtained in 159 patients (96.95 %; Fig. 2).

**Fig. 3:**
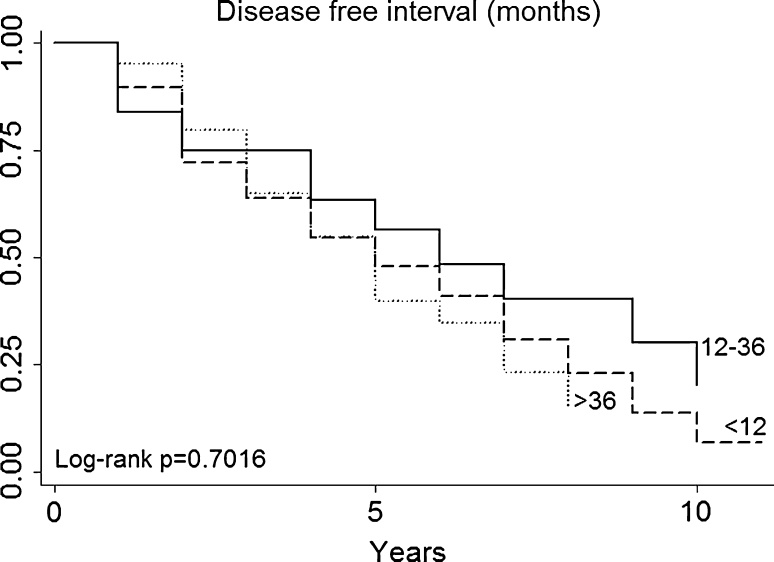
Histology

**Fig. 4:**
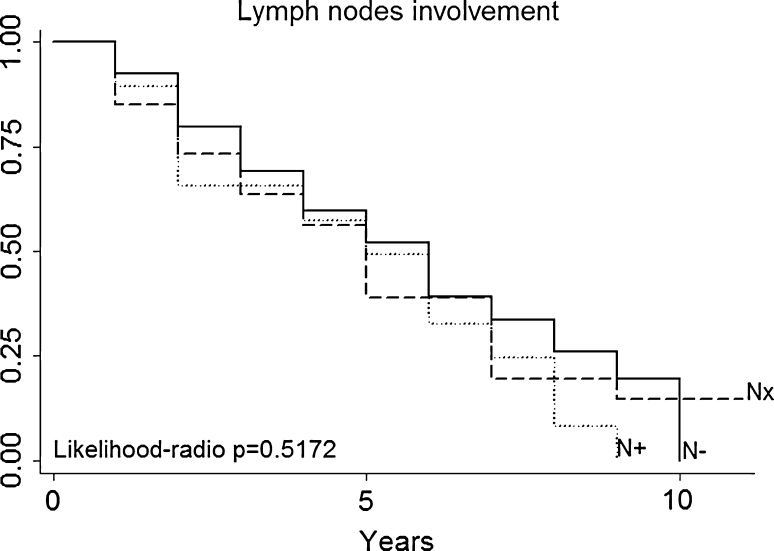
Disease Free Interval

One hundred patients (61 %) were men and 64 (39 %) were women. Mean age was 64 years (±14.4). Primary tumors were classified into four histological categories: epithelial, sarcoma, melanoma, and germ cell tumors. A total of 126 (77 %) patients developed metastases from epithelial tumors, 28 (17 %) developed from sarcoma, 7 (4 %) developed from melanoma, and 3 (1.8 %) from germ cell tumors (Table 1).

A total of 143 patients (87 %) underwent a single procedure (including six cases of bilateral planned thoracoscopic surgery within 30 days), and 21 patients (13 %) underwent multiple metastasectomies (redo surgery for 8 patients or contralateral surgery but later than 30 days for 13 patients). We performed a total of 212 metastasectomies with thoracoscopic lobectomy in 53 patients and thoracoscopic pneumonectomy in 1:136 wedge resections and 22 segmentectomies.

Nodal metastases were discovered in 20 cases (12 %); in 95 patients (58 %) nodes were negative. Node sampling was not performed in 49 (30 %) patients. All resection margins were tumor-free at final pathological examination.

We had no cases of local recurrence, defined as a tumor relapse at the site of the staple line. There were no intraoperative complications in any of the 164 VATS patients. The most common postoperative minor complications were arrhythmia, hydrothorax, pleural effusion, and air leak (Table 2).

**Table 2:** Minor postoperative complications

Arrhythmia	10
Pleural effusion	7
Prolonged air leak	7
Transfusion	6
Postobstructive pneumonia	3

Major complications occurred in four patients (2.4 %); two patients developed hemothorax (requiring one thoracoscopic drainage and the other a thoracotomy), two patients developed respiratory insufficiency and required admission to intensive care unit for 3 and 3 days. There were no postoperative deaths.

Conversion from VATS to thoracotomy occurred in 10 patients (6.5 %): four cases for pleural adhesion, four cases of diffuse metastatic disease, and two for mediastinal and hilar adenopathies.

There were no postoperative deaths, and the mean hospital stay after surgery was 5 days (interquartile range 4–8). One hundred twenty-three patients were admitted to the intensive care unit after the surgery with a mean stay of 1 day (range 1–5 days).

**Fig. 5:**
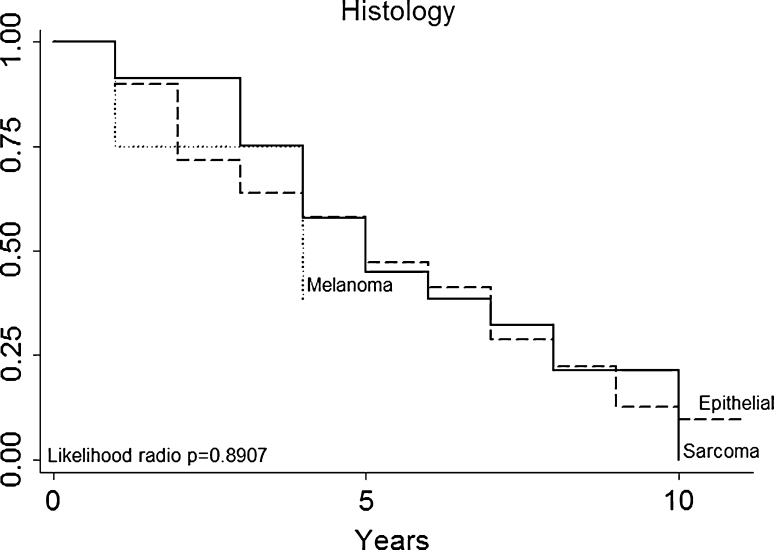
Nodal Status

**Fig. 6:**
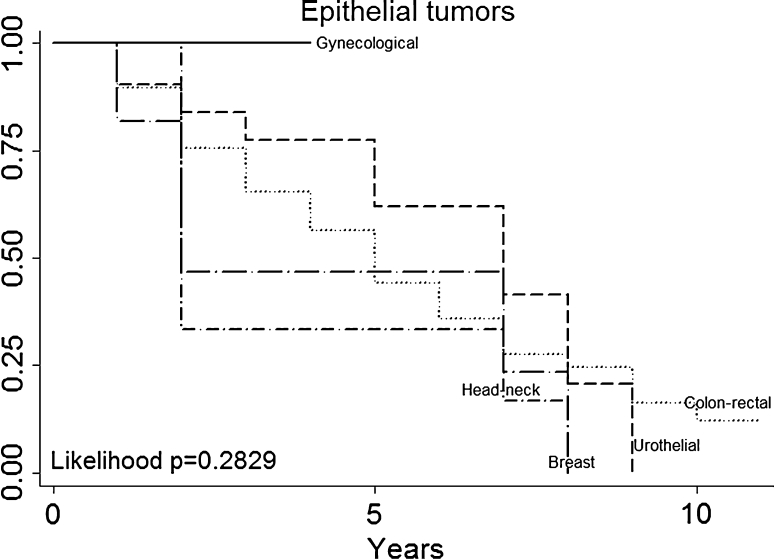
Epithelial tumors

**Fig. 7:**
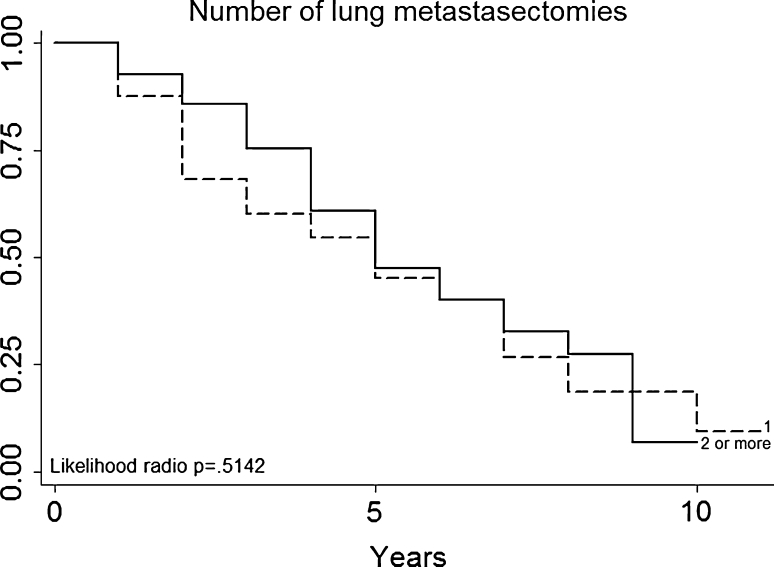
Number of metastasectomies

**Fig. 8:**
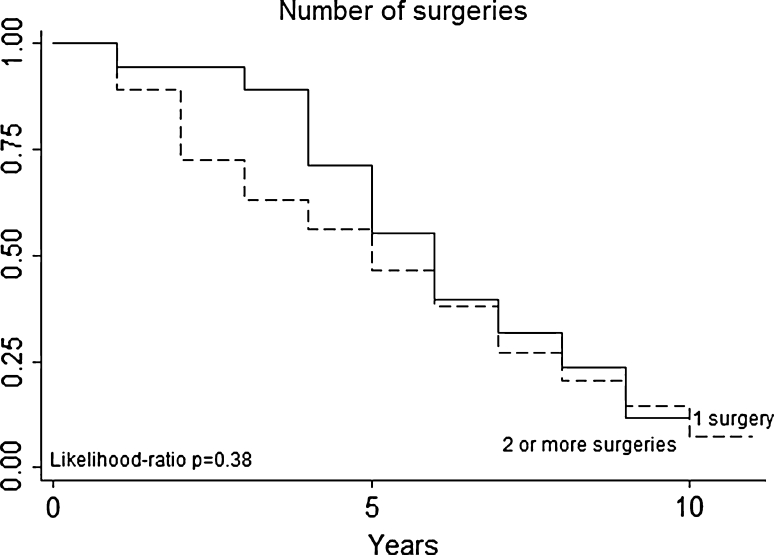
Number of surgeries

Multivariate analysis (Table 3) not confirmed in our series that patients with a particular histologic type (primary tumor histology, Fig. 3) had a better prognosis (for example, patients with melanoma or sarcoma in our series had no a worse prognosis than those with epithelial tumors). DFI (Fig. 4), nodal status (Fig. 5), epithelial tumors (Fig. 6), number of metastesectomies (Fig. 7), and number of procedures (Fig. 8) did not statistically influence long-term survival.

**Table 3:** Multivariate analysis for overall survival after pulmonary metastectomy

Variable	HR (95% CI)	*P*
Resection		
R0	0.53 (0.16–1.71)	0.29
R1	1	
DFI		
Simultaneous (mo)	1.25 (0.62–2.52)	0.52
1–12	1.00	
12–36	0.77 (0.4–1.46)	0.43
>36	1.06 (0.6–1.849	0.83
Sex		
Men	1	
Women	0.81 (0.51–1.28)	0.38
Age (years)		
<60	1	
60–69	0.61 (0.22–1.67)	0.33
70–79	0.90 (0.32–2.47)	0.83
>80	1	
Metastasis		
1	1	
2–3	0.74 (0.44–1.22)	0.24
>4	1.46 (0.68–3.12)	0.32
Histology		
Epithelial	1	
Sarcoma	0.9 (0.5–1.62)	0.74
Melanoma	1.46 (0.35–6.07)	0.59
Germ cell	1.11(0.34–3.61)	0.85
Surgery		
Wedge resection	1	
Lobectomy/segmentectomy	0.97 (0.61–1.53)	0.46
Nodal status		
Negative	1	
Positive	1.37 (0.74–2.53)	0.3
Unknown	1.13 (0.68–1.88)	0.63

**Fig. 9:**
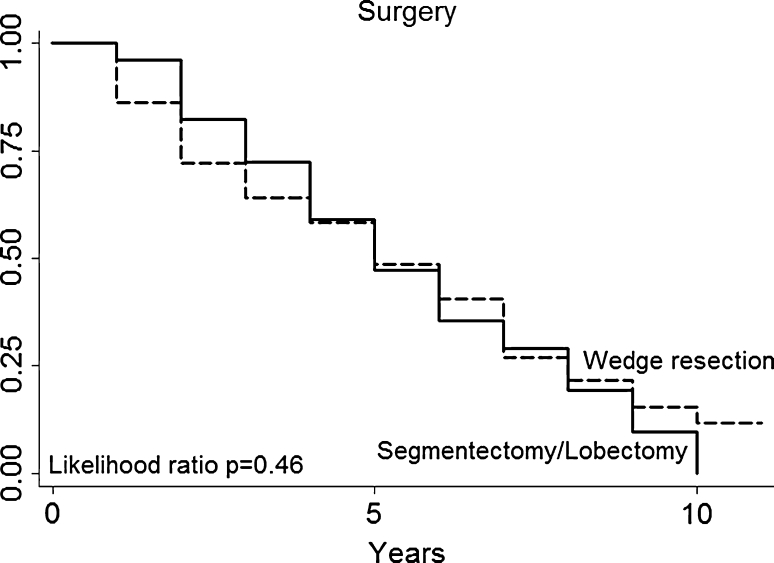
Surgery

Moreover, patients who underwent anatomical pulmonary resection (segmentectomy/lobectomy) compared with those who underwent wedge resection did not show any significant difference in long-term survival (Fig. 9).

## Discussion

The surgical approach to lung metastases remains highly variable. The choice of approach to pulmonary resection depends more on surgical training and personal conviction than on scientific basis. Certainly, the surgeon who operates on patients affected by advanced disease and history of pulmonary metastases has a moving target in terms of surgical approaches and systemic therapies [9]. Some authors prefer lung palpation during the operation to find and resect undetected lesions. According to previous reports, approximately 20 % of additional malignant nodules could be detected by manual palpation than by preoperative chest CT scan, but the clinical significance of these nonimaged, resected, malignant nodules is unknown [10, 11]. Other surgeons prefer minimally invasive surgery to perform pulmonary metastasectomy and VATS procedure can be performed with less morbidity and faster recovery. Potential advantages of the minimally invasive approach include smaller incisions, less postoperative pain, shorter length of stay, fewer adhesions at reoperation, and better compliance with adjuvant therapies if indicates. The initial data indicate that survival rates do not seem to significantly differ if the patients undergo VATS or open surgery [12–14]. Currently, there is no scientific evidence that resection of pulmonary metastases at the time they become radiographically apparent is any less efficacious than open procedures that remove all nodules, benign and malignant, before their radiologic identification. Besides, in open surgery there is the added morbidity of resecting benign nodules (approximately 50 % of nodules palpated), and the complete resection of all palpable metastatic nodules by open surgery is a not a complete biologic resection of all metastatic deposits [15].

Based on our experience, we believe that a surgeon with a large experience in VATS surgery has the ability to achieve complete resection; the ability of complete surgical excision of the metastatic nodules has been shown to be the most significant prognostic factor in the literature. This present study emphasizes the concept that in patients with lung metastases, the biology of the tumor is more important than the strategy of resection. In our thoracoscopic surgical experience, complete curative pulmonary resections were performed in 159 (96.95 %) cases and all resection margins were tumor-free at final pathologic examination. We had no a local recurrence (a tumor relapse at the staples site), and after a mean follow-up of 38 months, 77 (47 %) patients are alive and the overall survival is comparable with that of conventional open surgery. However, multivariate analysis in our study failed to identify any parameter that could statistically influence the long-term survival.

The recent development of multidetector row CT technology has made it possible to detect smaller-sized pulmonary nodules with excellent resolution during a single breath hold and to reconstruct CT images at a thickness of 1 mm. This new CT technology could improve the accuracy of CT scan in the detection of lung metastases and could be a substitute for manual palpation in pulmonary metastasectomy.

In case of recurrence of disease, a minimally invasive approach may be efficaciously repeated with low morbidity in the same patient, with a long-term outcome that is comparable with that after resection by thoracotomy. However, there is no evidence to suggest that the timing of resection of nodules that would be missed at thoracoscopic resection (which are usually <5 mm in diameter) is critical to the final outcome and, as we know, second resection do not adversely affect the overall outcome of patients with metasynchronous detected lesions. Thoracoscopic surgery may be favored in selected patients (for example those with a long DFI, or with a peripherally located nodule, or with several comorbidities, etc.), and we believe that the patient needs to be aware of all surgical options, with their advantages and limitations, and the best approach should be selected together with the surgeon.

For the future, there is need for prospective, clinical trials, performed in a multicenter setting, to obtain adequate answers about the best surgical approach for these patients, perhaps even with new CT technology use. Besides, is very important for the new generations of thoracic surgeons obtain an adequate training in thoracoscopic surgery, to ensure experience and familiarity also with a minimally invasive pulmonary metastasectomy.
